# Role of oxygen functional groups in reduced graphene oxide for lubrication

**DOI:** 10.1038/srep45030

**Published:** 2017-03-27

**Authors:** Bhavana Gupta, Niranjan Kumar, Kalpataru Panda, Vigneshwaran Kanan, Shailesh Joshi, Iris Visoly-Fisher

**Affiliations:** 1 Materials Science Group, Indira Gandhi Centre for Atomic Research, Kalpakkam, India; 2 Department of Solar Energy and Environmental Physics, Swiss Institute for Dryland Environmental and Energy Research, Jacob Blaustein Institutes for Desert Research, Ben-Gurion University of the Negev, Midreshet Ben-Gurion 8499000, Israel; 3 Department of Advanced Materials Science, Graduate School of Frontier Sciences, The University of Tokyo, 5-1-5 Kashiwanoha, Kashiwa, Chiba 277-8561, Japan; 4 Radiological Safety Division, Indira Gandhi Centre for Atomic Research, Kalpakkam, India

## Abstract

Functionalized and fully characterized graphene-based lubricant additives are potential 2D materials for energy-efficient tribological applications in machine elements, especially at macroscopic contacts. Two different reduced graphene oxide (rGO) derivatives, terminated by hydroxyl and epoxy-hydroxyl groups, were prepared and blended with two different molecular weights of polyethylene glycol (PEG) for tribological investigation. Epoxy-hydroxyl-terminated rGO dispersed in PEG showed significantly smaller values of the friction coefficient. In this condition, PEG chains intercalate between the functionalized graphene sheets, and shear can take place between the PEG and rGO sheets. However, the friction coefficient was unaffected when hydroxyl-terminated rGO was coupled with PEG. This can be explained by the strong coupling between graphene sheets through hydroxyl units, causing the interaction of PEG with the rGO to be non- effective for lubrication. On the other hand, antiwear properties of hydroxyl-terminated rGO were significantly enhanced compared to epoxy-hydroxyl functionalized rGO due to the integrity of graphene sheet clusters.

Lubricants reduce friction between contacting surfaces and thus increase the energy efficiency of moving machine components. Several remarkable properties of graphene-oxide/reduced graphene-oxide (GO/rGO) make it an effective solid additive for liquid lubricants^1^,^2^,^3^,^4^,^5^. These include low shear strength, high yield strength, chemical compatibility for dispersion in polar lubricants, and high thermal conductivity and an ecofriendly composition^5^,^6^. However, toxicity of graphene and related risks for the health cannot be ignored^7^. A small amount of GO/rGO is capable of effective lubrication due to high surface to volume ratio, effective inter-sheet shearing and sufficient oxygen functionality making GO/rGO compatible for dispersion in polar liquid lubricants^1^,^3^,^8^. The layered lattice structure is unique for reducing inter-sheet shear resistance and has shown low friction coefficients combined with the high strength of the graphene sheets^6^,^9^. Optimization of oxygen functionality in GO/rGO is an important aspect for preserving the chemical stability of the structure while providing sustainable covalent/noncovalent interactions with the lubricant. Lubrication enhancement by GO/rGO additives was described by the formation of low-shear-strength graphene tribofilm by adsorption to metal surfaces^1^,^2^,^10^. However, the lubrication mechanism was effective also without forming mechanically stable graphene tribofilms within the sliding interfaces^11^. In this case, lubrication acts through shearing of the physisorbed functionalized graphene sheets under the contact.

The properties of the liquid lubricant, including molecular weight and polarity, are also decisive for lubrication^12^,^13^. High molecular weight and high viscosity are important for high wear resistance while forming a high strength boundary tribofilm. However, shear resistance of the boundary film may increase the friction coefficient. Therefore, optimization of shear properties of the lubricant through the modification of viscosity is one of the most important criteria for effective lubrication. The dispersion of GO/rGO additives in the blends can assist in such optimization. For example, GO was also used as additive to enhance the antiwear properties of a mineral oil using a dispersant^2^. The dispersion of GO/rGO depends on the polarity of the lubricant and the functional groups of the GO/rGO. GO/rGO structures have a larger energy corrugation and higher shear strength than graphene due to localized electronic charge on the oxygen functionalities. These intrinsic properties degrade the tribological properties of GO/rGO compared to graphene. However, dispersion of graphene is not possible in hydrophilic lubricants due to the high cohesive energy, which makes the use of graphene for tribological applications unfeasible. The electrostatic and hydrogen bond interactions of GO/rGO and the dispersive medium were found to play a dominant role in its dispersion stability^14^,^15^,^16^. This is also related to the specific oxygen functionalization of GO/rGO. For example, a large lateral force is dominated by an interlayer hydrogen-bonded epoxy-hydroxyl coupling in GO/rGO. This leads to dissipation of frictional energy, manifested by higher adhesion and high strength of sliding resistance^17^. It should be noted that epoxy-epoxy and hydroxyl-hydroxyl interactions are weaker than epoxy-hydroxyl ones. The number of epoxide and hydroxyl groups in GO depends on the extent of oxidation of graphene. Higher oxidation of graphene may therefore induce more hydrogen bonds between epoxide and hydroxyl groups, indicating high binding energy and, consequently, higher shear strength^17^. However, the effects of hydroxyl and epoxy-hydroxyl GO functionalization on the micro/macrotribological properties of GO/rGO blended with a liquid lubricant have not been studied yet.

In this work, hydroxyl- and epoxy-hydroxyl-functionalized rGO samples were chemically prepared by controlled oxidation of bulk graphite powder followed by reduction. For simplicity, the hydroxyl- and epoxy-hydroxyl-functionalized rGO samples were designated as rGO_1_ and rGO_2_, respectively. The synthesized rGO was subjected to various microscopic and spectroscopic characterization techniques for detailed morphological, structural and chemical characterizations. The friction and wear properties of the rGO additives were investigated in two different molecular weights of polyethylene glycol (PEG). PEG is nonhazardous and it chemically interacts with rGO. Both rGO and PEG contain polar groups which provide effective dispersion stability through hydrogen bonding. Moreover, PEG is widely used in biomedical applications for reducing the friction and wear of knee joints^18^,^19^, but the use of rGO as blend additive to PEG for biomedical applications needs to be thoroughly investigated in term of safety^7^. The interaction mechanism of hydroxyl-hydroxyl and epoxy-hydroxyl terminated rGO with PEG were investigated. These interactions were used to interpret the friction and wear behavior of these rGO additives. The wear tracks were characterized by micro-Raman spectroscopy and X-ray photoelectron spectroscopy to reveal the friction and wear mechanism.

## Results and Discussion

### Chemical and structural characterization of rGO

**Figure 1:**
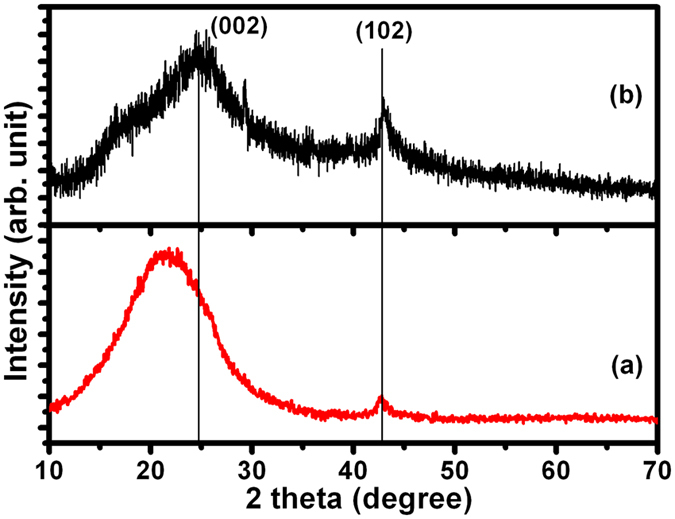
X-ray diffraction of (**a**) rGO_1_ and (**b**) rGO_2_ samples.

Well-resolved diffraction peaks of the (0 0 2) plane were observed in rGO_1_ and rGO_2_ at 2θ of 21.5° and 24.8°, respectively, indicating lattice spacing of 0.4 and 0.36 nm in rGO_1_ and rGO_2_, respectively (Fig. 1). In crystalline graphite, this value is 0.34 nm and the increase in lattice spacing in rGO indicates the presence of oxygen functional groups. The broad peak in these samples correspond to few layers of rGO sheets in the particles^20^, indicating the presence of multilayer domains along with few mono-layered rGO sheets. The shoulder at 2θ of 16.5° in rGO_2_ is related to intercalation of oxygen groups or water molecules causing increase in interlayer spacing to 0.53 nm^21^. A small diffraction peak at 2θ of 47.8° is related to the (1 0 2) plane of rGO structure.

**Figure 2:**
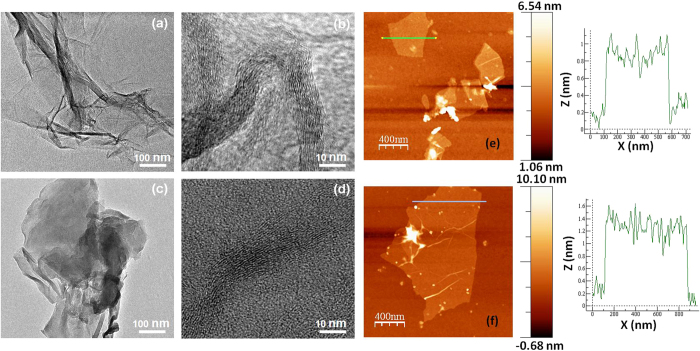
Low and high resolution TEM images of (**a**,**b**) rGO_1_ and (**c**,**d**) rGO_2_, and AFM images and the corresponding line profiles of exfoliated (**e**) rGO_1_ and (**f**) rGO_2_ samples.

The folded and corrugated morphology of the rGO sheets is shown in the low resolution TEM images (Fig. 2a and c). The surface corrugation is related to localized intrinsic strain due to loading of oxygen functional groups at the graphene sheets^6^,^22^. Well-aligned graphene/rGO sheets are evident in the high resolution TEM images (Fig. 2b and d). The AFM analysis showed rGO sheets of hundreds of nm in lateral dimensions and thicknesses of approximately 1 nm (Fig. 2e and f). This indicates that rGO contains few monolayers of graphene sheets in each particle, demonstrating the integrity of the graphene planes after the chemical treatments.

**Figure 3:**
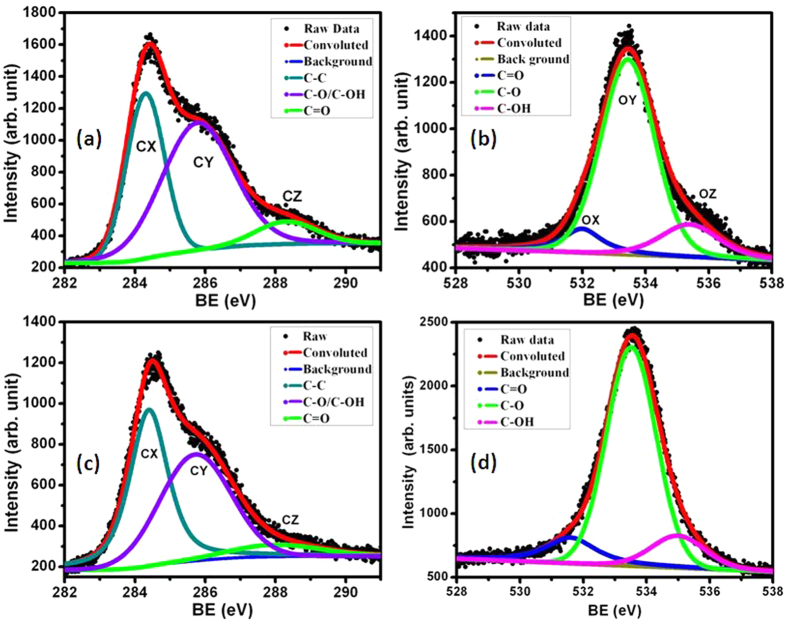
X-ray photoelectron emission spectroscopy of the C1s and O1s core levels of (**a**,**b**) rGO_1_ and (**c**,**d**) rGO_2_ samples, respectively.

In XPS, the C 1 s peak of both rGO samples was de-convoluted into three chemically shifted segments, designated CX, CY and CZ at binding energies of 284.3, 285.8 and 288.2 eV, respectively (Fig. 3a and c)^20^,^23^. Segment CX was assigned to non-oxygenated carbon of C–C/C–H representing the graphene structure^23^. This fact is well supported by XRD and TEM analysis. Segment CY described the interaction of carbon atoms with oxygen in either hydroxyl (C–OH) or epoxide (C–O) functional groups. The carbonyl (>C = O) segment was identified as CZ, which relates to carbon functionalized in COOH. The intensity of the carbonyl peak (CZ) was higher in rGO_2_ than in rGO_1_ samples, indicating a higher content of COOH complexes. This functional group is typically located at the edges of the graphene sheets, while the epoxy group evolves at the basal plane of the graphene sheets creating the in-plane defects and disorder^22^.

The ratio of the CX peak intensity to the sum of the (CY + CZ) peak intensities was smaller in rGO_2_ (0.72) than in rGO_1_ (0.84), indicating higher content of oxygen functionalities in rGO_2_. This is presumably associated with the higher concentration of the oxidizing agent KMnO_4_ used for the oxidation reaction of rGO_2_. The phenolic functional groups in rGO were investigated by de-convolution of the singlet O 1 s peaks into three individual segments, designated as OX, OY and OZ at 532, 533.4 and 535.4 eV, respectively (Fig. 3b and d). Segments OX, OY and OZ were assigned to C = O (oxygen double bond to aromatic carbon), C–O (oxygen single bond to carbon) and C-OH (carbon single bond to the hydroxyl group), respectively^20^,^23^. These peaks chemically shifted to 531.5, 533.2 and 534.9 eV in the rGO_2_ sample, i.e., slightly smaller binding energies, indicating a larger electronic charge on the oxygen atoms. This behavior further describes higher oxidation of rGO_2_. The OY/OX + OZ and OY/OZ ratios were larger in rGO_2_ (6.3 and 16.33) compared to rGO_1_ (4.23 and 7.2), indicating more carboxyl and carbonyl groups in the rGO_2_ sample. This result suggests denser epoxy network at the basal plane and a larger content of carbonyl linked at the edges of the graphene sheets in rGO_2_ sample compared to rGO_1_, associated with the harsher oxidation reaction in rGO_2_^20^,^22^.

**Figure 4:**
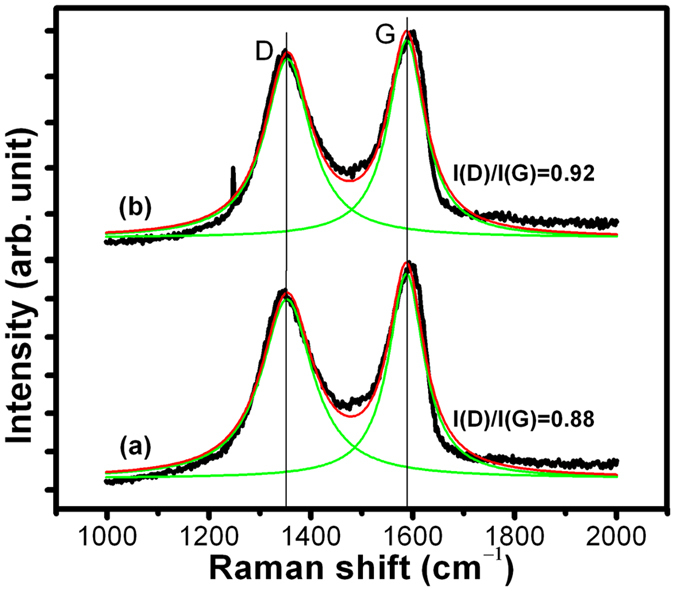
Raman spectroscopy of (**a**) rGO_1_ and (**b**) rGO_2_ samples with I(D)/I(G) ratios of 0.88 and 0.92, respectively.

In these samples, Raman spectra showed D and G bands at 1350 cm^–1^ and 1587 cm^–1^, respectively (Fig. 4). It is known that the D band arises due to A_1g_ symmetry and originates from the zone boundary phonons. This is attributed either to various defects or to a breakdown of translational symmetry in the graphite lattice. However, the G band is first-order scattering, related to E_2g_ symmetry and corresponds to the Brillouin zone of crystalline sp^2^ lattices in graphite^24^,^25^. The higher I(D)/I(G) ratio in the rGO_2_ sample indicates larger defect density (Fig. 4)^25^,^26^. Therefore, the Raman results indicate that the rGO_2_ contains a larger density of structural defects. The XPS results suggest that these defects are epoxy and carbonyl/hydroxyl functional groups at the basal plane and the edges of the graphene sheets, respectively. The larger defect is formed by unzipping of the carbon segments in the basal plane and developing epoxy network^22^. This network distorts the translational and periodic symmetry of graphite flakes.

**Figure 5:**
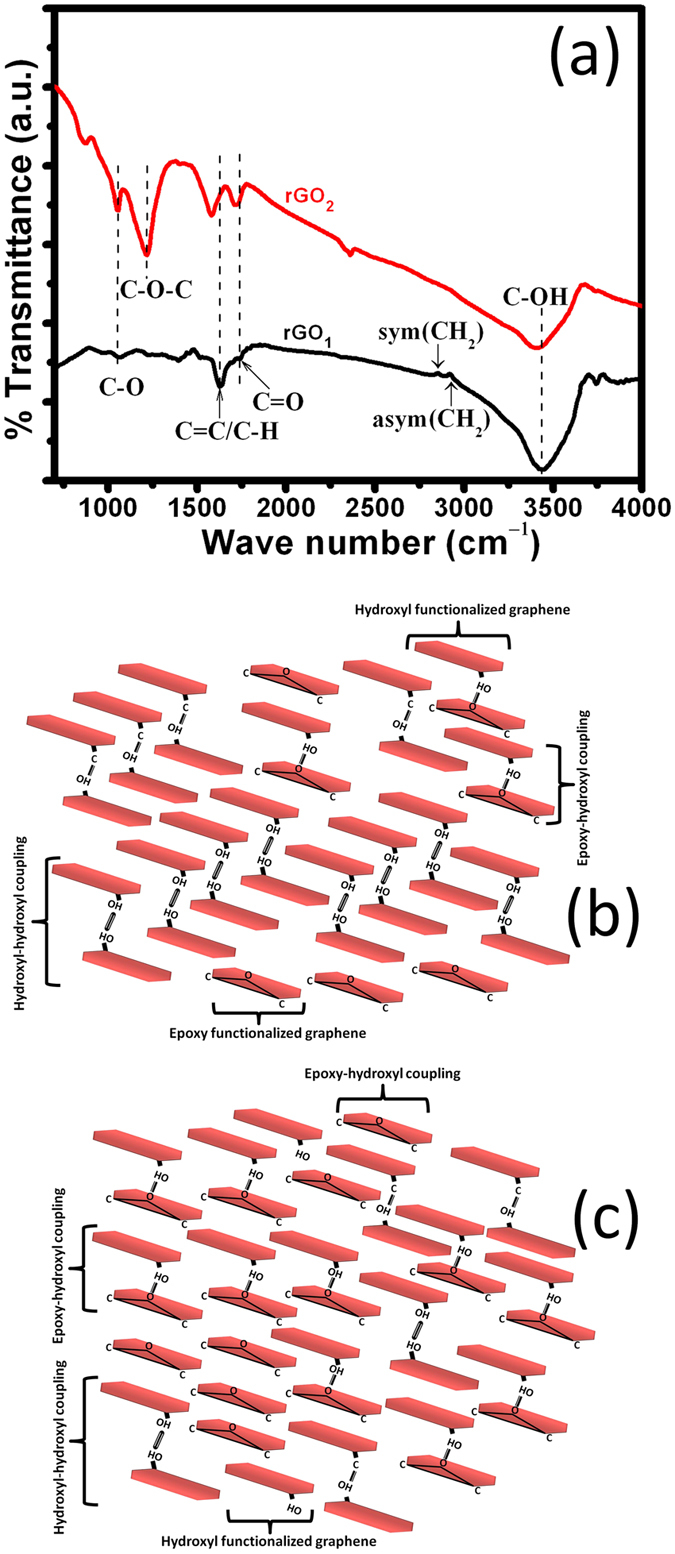
(**a**) FTIR spectra of rGO_1_ and rGO_2_samples, and schematic diagrams of (**b**) graphene sheets interacting through hydroxyl-hydroxyl groups in rGO_1_ sample, and (**c**) epoxy-hydroxyl terminated interaction of graphene sheets in rGO_2_ sample.

FTIR spectroscopy further revealed differences in functional groups between both types of rGO (Fig. 5a). In rGO_1_ sample, the –CH_2_ absorption band was split into two sub-bands centered at 2855 cm^–1^ (symmetric) and 2923 cm^–1^ (asymmetric) stretching modes, assigned to alkyl moieties (as a result of the reduction of COOH to -CH_3_ segment)^27^,^28^. These bands are very weak in these samples, indicating small amount of functionalized hydrogen moieties with carbon network.

A stretching band at 3435 cm^–1^ was related to the C–OH group and it was stronger in rGO_1_ sample, indicating a larger content of hydroxyl groups^20^. However, the absorption band intensities of –C–O (alkoxy) and –C–O (epoxy) groups at 1050 cm^–1^ and 1220 cm^–1^, respectively, were weaker in rGO_1_ sample. This is expected when concentration of oxidizing substances such as KMnO_4_ is not sufficient to break the strong covalently bonded carbon symmetry at the basal plane. In this condition, most of the oxygen groups will functionalize the edge of the graphene planes without producing lattice distortions and defects at the basal planes. The peak at 1650 cm^–1^ was attributed to the bending of O–H or C = C stretching, indicating stability of the graphene structure^27^,^28^. This peak was stronger in both the rGO_1_ and rGO_2_ sample and this is in agreement with the XPS data, indicating structural integrity. The shift of this peak to lower wave numbers in rGO_2_ sample may indicate stronger hydrogen bond coordination between the graphene-oxide sheets. This might be possible when hydroxyl-hydroxyl segments at the edge of the graphene sheets interact through hydrogen bonding^22^. Moreover, hydrogen bonding and electrostatic interaction between epoxy-hydroxyl units at the basal plane can also explain the peak shift, as hydrogen bond formation between C = O and O−H groups decreases the electron density of C = O due to electronic charge transfer from C = O to O−H antibonding orbital, thus lowering its vibrational frequency^28^. The FTIR analysis clearly indicates that rGO_1_ and rGO_2_ spectra were dominated by hydroxyl and epoxy-hydroxyl groups, respectively. Therefore, the coupling of the graphene-oxide sheets in rGO_1_ was expected to be facilitated via hydroxyl-hydroxyl interactions, mostly at the edges of the graphene sheets, as schematically shown in Fig. 5b. However, in the rGO_2_ sample, coupling of graphene sheets is expected to be predominantly through epoxy-hydroxyl interaction forming hydrogen bonding between the basal plane and edges of the graphene sheets (Fig. 5c). This fact is supported by the appearance of a shoulder at lower 2θ in XRD. In this sample, the presence of hydroxyl functional units at the edge of graphene sheets may also provide hydrogen bonding between networks of graphene sheets.

The chemical and structural characterization therefore indicates that rGO_1_ is mostly made of graphene sheets functionalized by hydroxyl groups at their edges facilitating inter-plane interactions. rGO_2_ is characterized by a larger density of oxygen-related functional groups, with many of them being epoxides residing on the graphene basal planes, in addition to hydroxyl groups, due to the harsher oxidizing conditions used in the manufacture of rGO_2_. Therefore, stronger inter-plane interactions are suggested in rGO_2_.

### Chemical characterization of PEG and rGO/PEG blends

The FTIR spectra of PEG200 and PEG600 showed a strong absorption band at 2870 cm^–1^, assigned to the –CH_2_ stretching vibration in alkyl chains (Fig. S1 in Supplementary Information). Peaks at 1353 cm^–1^ and 1498 cm^–1^ were ascribed to aliphatic C–H deformation^29^. The absorption band at 1062 cm^−1^ describes stretching vibration of the –C–O–C group attributed to the repeating oxyethylene (–OCH_2_CH_2_–) units of the PEG backbone. The band at 3410 cm^−1^ was ascribed to the stretching mode of C–OH in PEG200, which shifted to 3470 cm^−1^ and became weaker in PEG600 due to weak inter-molecular hydrogen bonding and smaller amount of hydroxyl units^28^,^29^,^30^. The increase in the ratio of –CH_2_/O–H stretching mode peak intensities in PEG600, indicate dispersive interactions between backbones of –CH_2_ units. However, in PEG200 intermolecular interactions are dominated by hydrogen bonding through hydroxyl-hydroxyl units. The wetting characteristics qualitatively describe the polarity of the molecules. This was studied by contact angle measurement, which showed a higher value (82°) for PEG600 than for PEG200 (65°) on steel substrates (Inset of Fig. S1). The enhanced wetting characteristic of PEG200 can be explained by the shorter chain length and the larger amount of hydroxyl end groups, which interact through hydrogen bonding with the polar metal-oxide surface containing hydroxides. Wetting properties also depends on the cohesive strength of molecules, which is stronger in PEG600 due to longer chain length and stronger dispersive forces, enhancing anti-wetting behavior.

The coupling of two different oxygen functionalized rGOs with two different types of PEGs, and the chemical interactions between these substances were investigated towards understanding the friction and wear mechanism. The preparation method of the samples for analyzing interaction between graphene oxide and PEG is provided below in the experimental methods section. This interaction was investigated by FTIR absorption spectroscopy and the results are shown in Fig. S2a. All FTIR absorption spectra showed a shift of the epoxy (C–O–C) vibrational mode to a higher frequency due to coordination of epoxy groups at the basal plane of graphene sheets with PEG molecules. In this case, the PEG molecules get intercalated in the basal plane gallery of epoxy-hydroxyl functionalized rGO_2_ sample (Fig. S2b). This is the appropriate reason for the appearance of shoulder peak in XRD at 2θ of 16.5° in rGO_2_ sample where interlayer spacing increased to 0.53 nm (Fig. 1). The similar behavior is shown by Wang *et al*.^27^. Most importantly, in these samples, the –CH_2_ bands at 2870 cm^–1^ and 2923 cm^–1^ becomes significantly stronger (Fig. S2) compared to neat rGO_1_ and rGO_2_ (Fig. 5). This clearly indicates coordination between alkyl moieties of PEG with graphene oxide samples (Fig. S2). The C = C and C–OH vibrational peak intensities were stronger and shifted to 1642 cm^–1^ and 3415 cm^–1^, respectively, when the rGO_1_ sample was functionalized with PEG200 and PEG600 (Fig. S2a spectra a_i_&b_i_). The absorption peak of C–OH was stronger in rGO_1_ functionalized by PEG200. In this sample, hydroxyl functionality dominated, and the coupling of graphene sheet and PEG molecules was expected through the hydroxyl and oxyethylene unit of graphene-oxide sheet and PEG^27^, respectively (Fig. S2c). Therefore, adsorption of PEG in the basal plane gallery is not expected in these samples, where epoxy units are not present on the graphene planes^22^. The interaction of PEG molecules with the oxygen functional group in the rGO_2_ sample showed a weak absorption band of the hydroxyl unit, but the absorption band of the epoxy group was stronger (Fig. S2a spectra c_i_&d_i_), as expected from hydrogen bonding between the epoxy unit of rGO_2_ and the hydroxyl moieties of the PEG molecules. This relation is schematically drawn in Fig. S2b. Reduction of the hydroxyl peak intensity in PEG600 blended with rGO_2_ is characteristic of PEG600. The peak at 1721 cm^–1^ was ascribed to the C = O stretching vibration mode in the carboxylic functional group (–COOH), indicating possible hydrogen bonding between PEG and graphene oxide^27^,^31^,^32^. This peak was stronger in the rGO_2_ sample blended with PEG200 and PEG600 due to large amount of carbonyl and carboxyl complexes, in agreement with the XPS analysis (Fig. 3).

The Raman spectra of the blended samples showed increased I(D)/I(G) ratio (Fig. S3) compared to pure rGO_1_ and rGO_2_ samples (Fig. 4). This clearly describes enhancement of structural defects, such as planer defects, staking faults, edges, vacancies, and bond distortions^24^,^25^,^33^ following blending with PEG. This ratio is higher in rGO_2_ blended additives and this could be explained by inherent high defect density in this sample (Fig. 4). Furthermore, it is important to notice that the I(D)/I(G) ratio is higher in PEG200 blended rGO_1_ and rGO_2_ samples than in PEG600 blends. The increase in defect density is related to effective coupling between rGO and PEG200 molecules. This could be related to the larger number of hydroxyl and oxyethylene units in PEG200, as indicated by FTIR analysis, due to their coupling with hydroxyl functional groups in rGO. The coupling of PEG600 with rGO might have been weaker also due to larger molecular weight, which makes inter-plane intercalation more difficult.

We conclude that the analysis indicates weaker interactions of PEG600 with both rGO types compared to PEG200 due to the smaller amount of hydroxyl content and larger inter-chain dispersive interactions in PEG600. For similar reasons, PEG200 shows better wetting of steel surface due to stronger interaction with polar groups on the steel surface. When blended with rGO_1_, PEG molecules interact only with the graphene plane edges via hydrogen bonding with the hydroxyl groups there. However, when blended with rGO_2_, PEG molecules intercalation between the graphene planes was indicated, mediated by the on-plane epoxide groups, in addition to hydrogen bonding with hydroxyl groups.

### Tribological properties

#### Tribological properties of neat PEGs

**Figure 6:**
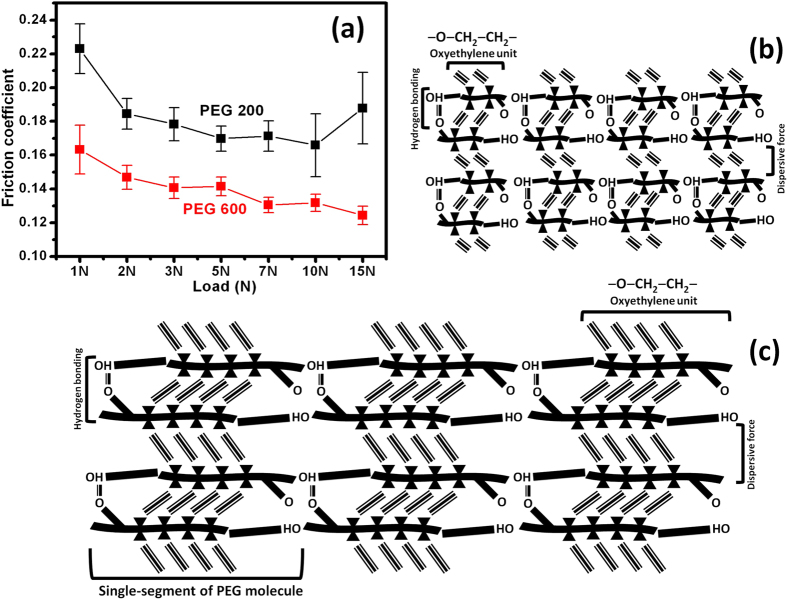
(**a**) Friction coefficient vs. normal load of neat PEG200 and neat PEG600, tribology test parameters: sliding speed: 4 cm/s, ball: 100Cr6 steel, sliding distance: 100 m. (**b**,**c**) Schematic description of the friction mechanism in PEG200 and PEG600, respectively.

The results of load dependent friction behavior of neat PEG200 and PEG600 are presented in Fig. 6a, and schematic diagrams of their molecular clusters are shown in Fig. 6b and c. *In*-*situ* detailed friction data are presented in Fig. S4. These experiments served as controlled experiments and helped to select load points for comparison with rGO-blended PEGs. The friction coefficient of PEG600 was found to be lower than that of PEG200, and for both lubricants, this value decreased with increasing normal loads, which is explained by the shear-thinning of lubricant layer and a decrease in the intermolecular friction in the confined volume^34^. An increase in the friction coefficient at 15 N loads in PEG200 was observed. This may have been caused by a critical reduction of the lubricant film thickness in the contact, where critical boundary lubrication regime gives rise to shear resistance between the molecular segments of PEG. In this condition, the role of the lubricant becomes ineffective, while friction between solid metal surfaces dominates^35^,^36^. However, the viscosity of PEG600 is higher than that of PEG200 due to larger intermolecular dispersive forces (see Fig. S1 in Supplementary Information and related discussion), causing resistance to boundary-lubrication condition, hence no increase in friction coefficient was noted at higher loads.

The friction coefficient of PEG600 was found to be smaller than that of PEG200 throughout the entire measured load range. Intermolecular shear resistance in liquid generally increases with the viscosity, as expected for PEG600 (see above)^26^,^28^. An increase in the shear modulus of high molecular weight PEG was confirmed by Ping Wang *et al*.^37^. For larger PEG molecules, polymer coils resist the shear stress, which appreciably contributes to the shear modulus. The PEG600 should show higher friction coefficient due to high viscosity, increased internal friction and high shear modulus, however the results showed a different behavior. The reasons for the lower friction coefficients of PEG600 might include increase of inter-molecular repulsive forces in stressed conditions due to a decrease in the inter-molecular distance of methylene units. The reduced friction coefficient might also be related to reduced shear due to directional degree of freedom of longer methylene units in PEG600 and the smaller density of cross-linking hydrogen bonded functional groups^12^,^38^,^39^,^40^,^41^,^42^, as schematically shown in Fig. 6c. The hydroxyl groups of the PEG also interact through hydrogen bonding with hydroxides present at the steel surface^40^. This is the reason for the decrease in contact angle of PEG200, indicating better affinity to the steel surface, as discussed above with respect to FTIR and contact angle analysis. This could also explain the higher friction coefficients of PEG200.

#### Friction properties of PEG/rGO blends

**Figure 7:**
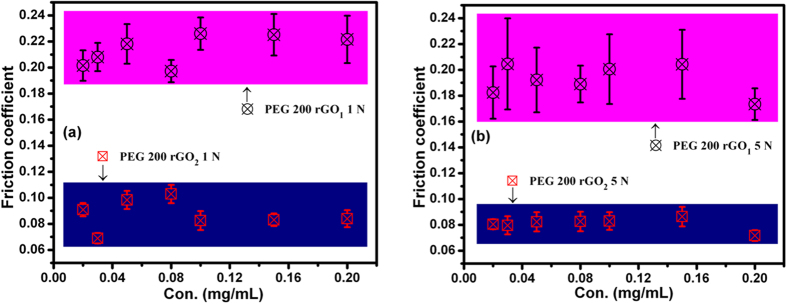
Friction coefficient vs. concentration of rGO_1_ and rGO_2_ additives in PEG200 at loads of (**a**) 1 and (**b**) 5 N; tribology test parameters: sliding speed: 4 cm/s, ball: 100Cr6 steel, sliding distance: 100 m.

**Figure 8:**
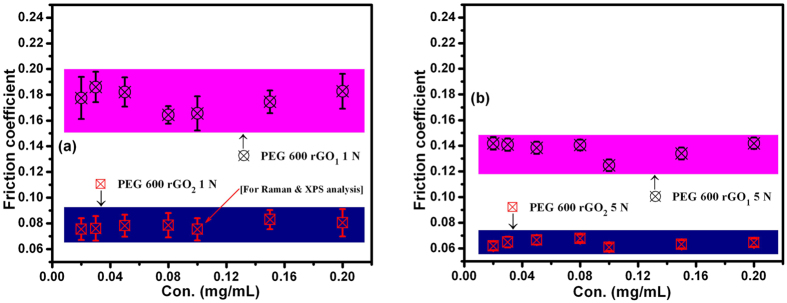
Friction coefficient vs. concentration of rGO_1_ and rGO_2_ additives in PEG600 at loads of (**a**) 1 and (**b**) 5 N; tribology test parameters: sliding speed: 4 cm/s, ball: 100Cr6 steel, sliding distance: 100 m.

The friction results of the rGO_1_ and rGO_2_ samples as a function of the additive concentration in PEG200 and PEG600 at 1 N and 5 N are shown in Figs 7 and 8. Detailed *in*-*situ* friction data is given in Figs S5–S8.

**Figure 9:**
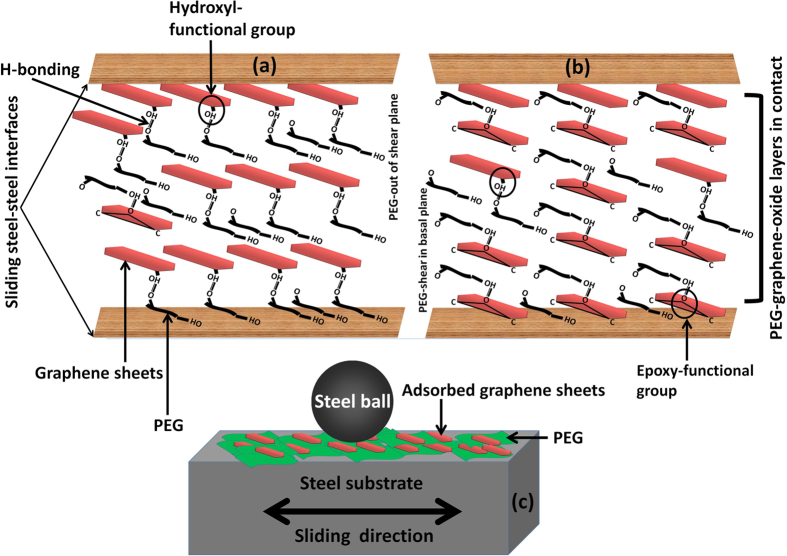
Schematic description of the friction mechanism in (**a**) hydroxyl- (rGO_l_) and (**b**) epoxy-hydroxyl- (rGO_2_) functionalized rGO additives blended with PEG. Edges of the graphene sheets interact with PEG in hydroxyl terminated rGO_1_, however epoxy-hydroxyl groups at edges and on-plane of the graphene sheets in rGO_2_ both interact with PEG yielding efficient PEG intercalation. PEG blended graphene-oxide sheets formed boundary tribofilm in sliding contact (**c**), and then these sheets get adsorbed on the sliding surface forming a low shear strength tribolayer.

The friction coefficient of blends of rGO_1_ and rGO_2_ in PEG600 were lower than those of blends with PEG200, in accordance with the neat PEG properties discussed above. rGO_1_ blended in PEG200 and PEG600 showed higher values of the friction coefficient than rGO_2_ under both loading conditions (Figs 7 and 8), with values comparable to those of neat PEGs at these loads (Fig. 6a). However, rGO_2_/PEG blended samples showed significantly smaller friction coefficients. These observations could be directly related to the types of functional groups available in rGO samples, and to the chemical properties of the two different PEGs, both affecting the coupling of functionalized rGO sheets with PEGs. Hydroxyl and epoxy-hydroxyl groups were found to be dominant in the rGO_1_ and rGO_2_ samples, respectively (see above). The principal interaction mechanisms in the rGO_1_ sample include electrostatic attraction between the oxygen atom in one layer and the carbon atom of the C–O moieties in an adjacent layer^17^, and hydroxyl-hydroxyl interactions through electrostatic forces. The primary interaction mechanism observed in the rGO_2_ sample was the epoxy-hydroxyl interaction via hydrogen bonding (Fig. 5). The binding energy was stronger in the latter type of interaction^17^. Wang *et al*.^17^ have shown that epoxy-epoxy and hydroxyl-hydroxyl interactions between functionalized graphene sheets induced a smaller critical sliding force than hydroxyl-epoxy interactions. In this regard, the friction coefficients should have been lower in the hydroxyl-dominated graphene sample, i.e., rGO_1_, but the results of the rGO/PEG blends showed the opposite. However, in rGO_1_/PEG blends, the PEG was coupled through the hydroxyls at the graphene edges and the oxygen of the oxyethylene units in PEG. This kind of coupling did not contribute to decreasing in shear resistance because the PEG molecule did not intercalate between the graphene planes^18^. The friction mechanism in hydroxyl-dominated graphene oxide (rGO_1_) sample is schematically shown in Fig. 9a. Here, edge functionalized graphene-oxide sheets interact with PEG and may lubricate the steel-steel contacting interfaces. In contrast, rGO_2_ was predominantly functionalized by the epoxy groups, allowing coupling of hydroxyl units of the PEG with oxygen of the epoxy units at the basal plane in rGO_2_ sample. Inter-layer shear of the graphene sheets was enabled by the intercalated PEG molecules, explaining reduction of the shear resistance. This is schematically presented in Fig. 9b. In both rGO_1_ and rGO_2_ blends the friction coefficients were almost concentration independent (Figs 7 and 8). Therefore, an extremely small amount of rGO_2_, e.g. 0.02 mg/mL, is sufficient to effectively lubricate the steel-steel sliding contact. The contact condition of linear reciprocating sliding mode is shown in Fig. 9c.

Here, PEG blended rGO sheets formed a boundary tribofilm in the tribo- contact and these are represented in Fig. 9a and b. Under the continuous sliding motion, these sheets get adsorbed at the surface of metallic tribo- contact, forming permanent and sustainable boundary tribofilm (Fig. 9c). The evidence of tribolayer is characterized in the later section.

#### Wear properties of PEG/rGO blends

**Figure 10:**
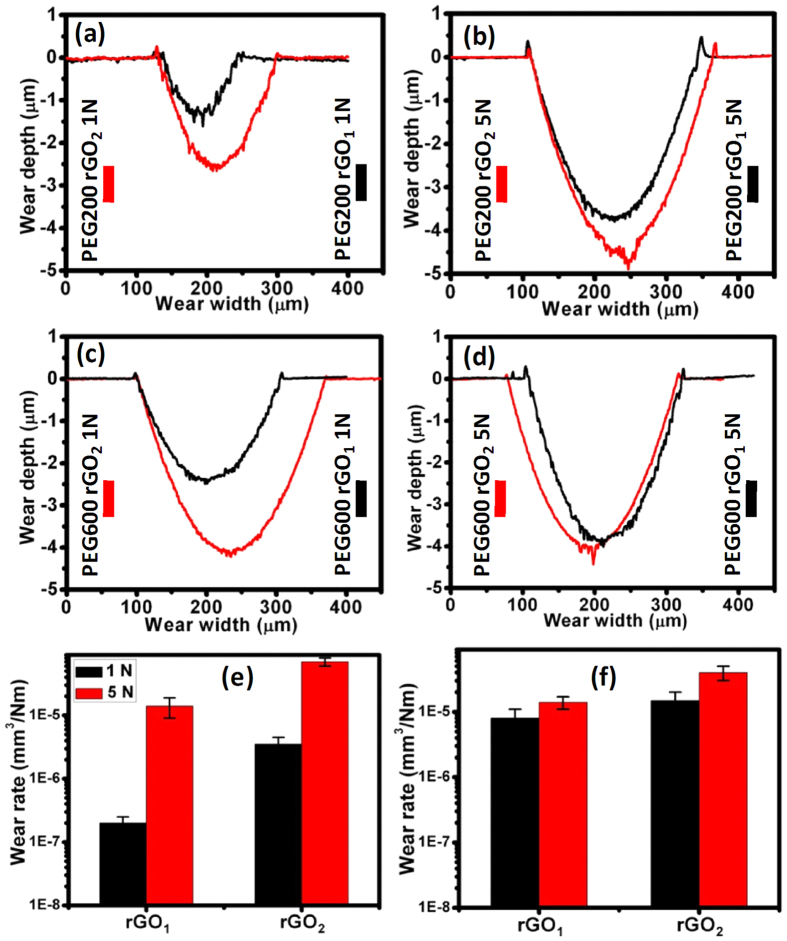
2-dimensional wear loss analysis of: (**a** and **b**) rGO_1_ and rGO_2_ dispersed in PEG200 at 1 and 5 N, respectively, (**c** and **d**) rGO_1_ and rGO_2_ dispersed in PEG600 at 1 and 5 N, respectively, and calculated wear rate in blends in (**e**) PEG200 and (**f**) PEG600. Tribology test parameters: concentration: 0.1 mg. mL^–1^, sliding speed: 4 cm/s, ball: 100Cr6 steel, sliding distance: 100 m.

Wear track analysis showed significantly smaller wear track width in PEG200 compared to PEG600 due to the strong wetting of PEG200 on steel surface (see above) that forms a high strength boundary film, minimizing the metal-to-metal contact which enhanced the antiwear properties (Fig. S9a). Selective wear tracks were subjected to 2-dimensional quantitative analysis and wear rate calculations. For this purpose, wear tracks formed at 1 N and 5 N loads with blends with 0.1 mg. mL^–1^ rGO_1_ and rGO_2_ additives with PEG200 and PEG600 were selected. The wear tracks and ball scars impression are presented in Fig. S10a–h. Highly effective antiwear behavior was observed in both rGO_1_ and rGO_2_ blended PEG200 lubricant at 1 N load (Fig. 10a), where the smallest damage of sliding bodies was observed in optical images (Fig. S10a and b). However, the wear loss was higher in rGO_1_ and rGO_2_ blended PEG600 at similar tribo-test conditions (Fig. 10c). Furthermore, at higher loads (5 N), both sets of samples lost the antiwear properties (Fig. 10b and d), similar to the neat PEG behavior (Fig. S9a). Therefore, it is clear that antiwear property of PEG200 is intrinsic characteristic of the PEG molecules. No clear relationships of concentration-dependent wear resistance were observed in both the additives blended PEGs, but the wear loss increased at high loads (Fig. S9c and e). This is clearly observed in 2-dimensional profiles of the wear tracks (Fig. 10b and d). This might be explained by smaller thickness of the lubricant layer in the sliding interfaces due to higher contact stress. In this condition, boundary lubrication operates where direct metal to metal contact dominates.

Moreover, results showed smaller wear track dimensions in the case of rGO_1_-compared to rGO_2_- blended in both PEG200 and PEG600. This is associated with intrinsic characteristics of hydroxyl terminated rGO sample which effectively protect against wear. It was noticed that antiwear performance was extremely good in the rGO_1_ sample at 1 N load (Fig. 10a), but the friction coefficient was very high under similar tribo-condition (Figs 7 and 8). In contrast, the wear track dimension was roughly doubled in the rGO_2_ sample at similar tribology conditions. In all the tribo-test conditions, the rGO_1_ sample was superior for wear resistance (Fig. 10). Calculation of wear rate also clearly showed high wear resistance of rGO_1_ sample (Fig. 10e). This calculation was carried out using the simple relation, *k* = W/F.s, where *k*, W, F and s are the wear rate, wear volume, normal force and sliding distance, respectively. Improved wear resistance properties of the blends could result from two factors: (a) hydrophilic properties of PEG200, which inherently protects the surface against wear forming boundary film. This is independent of the chemical properties of rGO_1_ and rGO_2_, and (b) rGO_1_ is hydroxyl functionalized and the compressive strength of such a functionalized structure is higher than that of rGO_2_. This is because rGO_1_ functionalization creates few defects on the plane of the graphene sheets as compared to epoxy functionalization of rGO_2_^22^,^43^, as hydroxyl groups mostly occupy the edges of the graphene sheets^22^, in agreement with Raman and FTIR analysis (Figs 4 and 5). Tribology results of neat rGO_1_ and rGO_2_ sample without PEG are presented in Fig. S11 and it showed extremely low frictional coefficient in rGO_1_ sample, as expected. It can therefore be concluded that PEG200 and rGO_1_ blends provide the best anti-wear properties due to favorable combination of the constituents’ properties.

#### Spectroscopic analysis of wear tracks

**Figure 11:**
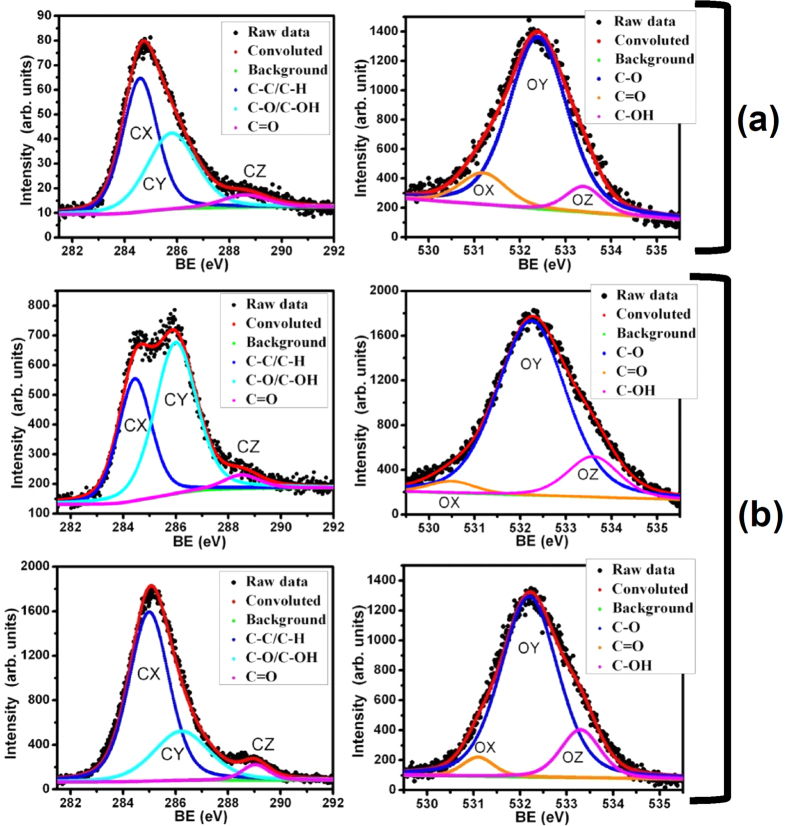
X-ray photoelectron emission analysis of carbon (C 1 s) and oxygen (O 1 s) chemical shifts (**a**) at the steel surface and (**b**), at the surface of the wear track at various locations. Tribology test parameters: rGO_2_ sample, concentration: 0.1 mg/mL, load: 1 N, sliding speed: 4 cm/s, ball: 100Cr6 steel.

Decrease in friction coefficient and high wear resistance properties could be traditionally explained by deposition of the graphene sheets at the tribo-contact region^1^,^10^. The presence of these sheets in the contact interfaces are directly related to dispersion stability and chemical interaction of the functionalized graphene sheets with the lubricant oil. The interaction aspect of functionalized graphene sheets with the lubricant is shown in Fig. S2 and the friction mechanisms are schematically drawn in Fig. 9. Hence, a chemical analysis of the wear track was carried out by Raman and XPS to provide evidence of tribolayer. Micro-Raman spectra of the wear track mainly showed a signature of metal-oxide and carbon peaks (Fig. S12). A very weak and diffused carbon signal in the wear track indicated the amorphous and elemental nature of carbon, which is inherently present (0.03%) in the steel substrate (316 LN). This was confirmed by the Raman spectra obtained from the steel surface (Fig. S12, spot (a)) of the same material, which showed a similar type of Raman shift to that of the wear track (Fig. S12, spots (b-d)). Therefore, evidence of carbon deposition from the rGO blended lubricant on the surface was not provided by Raman analysis. It is important to note that Raman spectroscopy is not a surface sensitive technique and chemical signals are mostly obtained from the subsurface region. However, XPS spectra at different locations of the same wear track showed prominent features of carbon and oxygen species (Fig. 11b). In these spectra, three photoelectron emission bands, i.e., segments C–C/C–H (284.4–284.8 eV), C–O/C–OH (285.6–286 eV) and C = O (287.5–289 eV)^20^,^23^ were designated as CX, CY and CZ, respectively. The chemical shifts at the wear track surface showed similar photoelectron emission characteristics to those shown of rGO_2_ sample (Fig. 3). The intensity of the C–O/C–OH peak in the wear track was enhanced compared to the virgin rGO sample. This might have been caused by the tribo-induced structural disordering of the graphene, and/or to oxidation of the graphene sheets during the tribology test. This evidence supports the deposition of chemisorbed rGO sheets on the surface of the wear track. The chemical shifts designated by the CX, CY and CZ segments were also present at the virgin steel surface, but their intensity was significantly weaker under similar XPS experimental conditions (Fig. 11a). The presence of oxygen species deduced from the O 1 s spectra in the virgin steel surface was also compared with that at various locations on the wear track (Fig. 11b).

Segments OX, OY and OZ in the wear track are associated with the interaction of carbon with oxygen species in various chemical configurations and oxidation states. The shapes and chemical shifts of these photoelectron emission peaks are quite similar to the as-prepared rGO samples (Fig. 3). However, the relative intensity of the OZ segment was increased in the wear track compared to the steel surface, indicating the presence of rGO moieties. From the spectroscopic results, it is concluded that the deposition of oxidized carbon species in the wear track has occurred, though a contribution of carbon inherently present in the steel surface cannot be ruled out. These observations confirm that graphene/graphene-oxide moieties are adsorbed on the surface of the wear track and participate in the lubrication process.

## Conclusions

Two different reduced graphene oxide (rGO) derivatives were prepared for tribological investigation and blended with two different molecular weights of polyethylene glycol (PEG). Chemical and structural characterization indicated that rGO_1_ is mostly made of graphene sheets functionalized by hydroxyl groups at their edges facilitating inter-plane interactions. rGO_2_ is characterized by a larger density of oxygen-related functional groups, with many of them being epoxides residing on the graphene basal planes, in addition to hydroxyl groups, due to the harsher oxidizing conditions used in the synthesis of rGO_2_. Weaker interactions were found for PEG600 with both rGO types compared to PEG200 due to the smaller hydroxyl content and larger inter-chain dispersive interactions in PEG600. When blended with rGO_1_, PEG molecules interact only with the graphene plane edges via hydrogen bonding with the hydroxyl groups there. However, when blended with rGO_2_, PEG molecules intercalation between the graphene planes was indicated, mediated by the on-plane epoxide groups, in addition to hydrogen bonding with hydroxyl groups.

The friction and wear properties of PEG with two different molecular weights, namely PEG200 and PEG600, were compared in steel-steel contact interfaces. The higher molecular weight of PEG600 showed a lower friction coefficient, explained by the longer methylene-chain unit and enhanced anti-wetting properties, indicating effective boundary film lubrication. PEG200 showed higher values of the friction coefficient due to a strong wetting of the sliding interfaces. These results were compared with the tribological properties of PEG blends with the two different rGO additives functionalized by hydroxyl and epoxy-hydroxyl groups. Epoxy-hydroxyl functionalized rGO_2_-containing blends showed significantly lower friction coefficients compared to hydroxyl-terminated rGO_1_ additives blended in both types of PEGs. This was explained by intercalation of PEG molecules between the basal planes of the graphene sheets through epoxy-hydroxyl interactions between rGO_2_ and PEG. This decreased shear resistance of the sliding sheets and further reduced the frictional energy at the steel-steel interfaces. An extremely small concentration of rGO_2_, e.g. 0.02 mg/mL, was found to be sufficient to effectively lubricate the steel-steel sliding contact.

The mechanism of friction is well explained by adsorption of graphene/graphene-oxide sheets in the tribo-contact region which act as low shear strength boundary film, as demonstrated by XPS analysis performed in the various locations of the wear track. The antiwear properties were superior in hydroxyl-terminated rGO_1_. This was explained by a smaller defect density induced by the functional groups in the planes of the graphene sheets, which preserved the mechanical integrity of the graphene clusters to sustain the load bearing capacity of graphene-oxide boundary film. This kind of lubricant additives is easy to prepare and may be suitable for various potential applications. We summarize that rGO_1_ reduces wear but increases friction, while rGO_2_ reduces friction but does not effectively reduce wear.

## Experimental Methods

### Synthesis of hydroxyl-terminated rGO (rGO_1_)

The sample was prepared by Hummer’s method^44^,^45^. Graphite flakes (10 g) and NaNO_3_ (10 g) were mixed in 500 mL of H_2_SO_4_ (98%) at 5 °C with continuous stirring for 2 h, and KMnO_4_ (30 g) was gradually added to the suspension at a controlled reaction temperature lower than 15 °C. The ice bath was then removed, and the mixture was stirred at 35 °C until it became pasty brownish; stirring continued for two days, after which the mixture was diluted with a slow addition of 300 ml of water. The reaction temperature was rapidly increased to 98 °C, and the color changed to dark brown. This solution was further diluted by adding an additional 400 ml of water and stirring continuously. The solution was finally treated with 150 ml of H_2_O_2_ to terminate the reaction, indicated by the appearance of a yellow color. For purification, the mixture was washed by rinsing and centrifugation with 10% HCl and then with deionized water (DI) several times. After filtration and drying under a vacuum at room temperature, the GO_1_ was obtained as powder. Reduction was performed to reduce the functional group density, towards decreasing the inter-plane spacing and the flake dimensions and improving the friction properties. Reduction was done via treating 100 mg of GO_1_ in 30 mL of water with 3 mL of hydrazine and refluxing at 95 °C for 24 h, followed by filtering, washing and drying at 60 °C under vacuum to obtain the rGO_1_ powder.

### Synthesis of epoxy-hydroxyl-terminated rGO (rGO_2_)

Graphite flakes (10 g) and NaNO_3_ (10 g) were mixed in 600 mL of H_2_SO_4_ (98%) at 5 °C with continuous stirring for 6 h, and KMnO_4_ (60 g) was gradually added to the suspension while maintaining the temperature at 15 °C. The mixture was diluted with a slow addition of 400 mL of water and with stirring maintained for 2 h. The ice bath was then removed, and the mixture was stirred at 35 °C for 2 h and then refluxed at 98 °C for 30 min. The solution was finally treated with 200 ml of H_2_O_2_ and the color changed to bright yellow. The resulting mixture was washed by centrifugation with 10% HCl and then with DI water several times until it became gel-like. After centrifugation, the gel was vacuum dried at 80 °C for 8 h to obtain the GO_2_ powder. Reduction was performed similarly to rGO_1_.

### PEG and rGO-PEG blends

Two different molecular weights of PEG200 and PEG600 (from Sigma Aldrich) were used as synthetic lubricating base oils for tribology evaluation of the rGO additives. The average number of oxyethylene units in PEG200 is approximately 4 and its viscosity is 8.5 cSt. Viscosity of PEG600 is 18 cSt, and the average number of oxyethylene units is 13. The higher viscosity is caused by stronger intermolecular dispersive forces. Both rGO samples were dispersed and ultrasonicated for 3 h with a concentration of 1 mg/mL in PEG200 and PEG600. The samples were centrifuged, thoroughly washed in ethanol and DI water, and heat treated at 130 °C for 8 h to remove physisorbed PEG, ethanol and water molecules. Chemical analysis was applied to the dry powder samples of PEG200 and PEG600 functionalized by rGO_1_ and rGO_2_.

### Characterization techniques

The surface topography of the rGO samples were analyzed by atomic force microscopy (AFM, Keysight 5500). For AFM analysis, the rGO samples were ultrasonicated in ethanol for 2 h, resulting in partial exfoliation. The flakes were deposited on silicon substrates that were kept inside the ethanol bath during ultrasonoication. Elimination of the oxygen functionalities from the rGO was the main reason for exfoliation. The microstructure and crystallography were further investigated by high resolution transmission electron microscopy (HR-TEM) and XRD pattern was obtained using a PAN-alyticalX’pert Pro dual goniometer consisting of a CuKα radiation source (λ = 0.15418 nm). The chemical composition was investigated by XPS (ULVAC-Phi ESCA1800), using a monochromatic MgKα source (400 W, 1253.6 eV) and by a micro-Raman spectrometer (Ranishaw) operating at a laser wavelength of 514.5 nm, which was also used for tribofilm analysis. An FTIR spectrometer (Bruker Optics), operating in transmission mode with a spectral resolution 4 cm^−1^, was used for the analysis of functional groups. Contact angle measurements of PEG200 and PEG600 on a steel surface were carried out using a contact angle meter (Holmarc, HO-IAD-CAM-01, India) equipped with a CCD camera. The volume of the droplet used for the contact angle measurement was approximately 1 μL.

### Tribology tests

Before the tribology test, the rGO was thoroughly dispersed in PEG by ultrasonication, and its dispersion stability was monitored. The tribological properties of the rGO dispersed in PEG were studied by measuring friction and wear at various loads, and rGO concentrations were measured using a ball-on-disc standard tribometer (CSM Instrument, Switzerland), operating in a linear reciprocating mode. A 100Cr6 spherical steel ball with a diameter of 6 mm was used to slide against a lubricated steel (316 LN) disc at a sliding speed of 4 cm/s. The surface roughness values of the ball and disc were approximately 32 and 17 nm, respectively. In each test, two drops of the ultrasonicated PEG blended with the rGO lubricant were used to thoroughly lubricate the sliding interfaces. A more detailed tribological experimental procedure is described elsewhere^3^,^10^. Two dimensional wear track profile were measured by a Dektak 6 M–stylus profiler in contact mode, at fixed 5 mg contact load and 10 μm/s scanning speed. In this method, the tip of the diamond stylus was scanned across the wear track generating 2D wear profile. All the tribological experiments and wear measurements were generally conducted four to five times and the error bars was represented with data. The reproducibility of the test was excellent and the value for error deviation was marginal. The stylus is mechanically coupled to the core of an LVDT sensor. Wear track image was obtained by optical microscope for damage and wear width analysis. Before the XPS analysis, the wear track was ultrasonicated in deionized water and dried at 110 °C to remove physisorbed contaminating particles.

## Additional Information

**How to cite this article:** Gupta, B. *et al*. Role of oxygen functional groups in reduced graphene oxide for lubrication. *Sci. Rep.* **7**, 45030; doi: 10.1038/srep45030 (2017).

**Publisher's note:** Springer Nature remains neutral with regard to jurisdictional claims in published maps and institutional affiliations.

## Supplementary Material

Supplementary Information
