# Supplementary material for: Role of oxygen functional groups in reduced graphene oxide for lubrication
Source: Sci Rep. 2017 Mar 27;7:45030. doi: 10.1038/srep45030 (PMC5366868; doi:10.1038/srep45030)
Supplement: Supplementary Information [file srep45030-s1.pdf]

Supporting Information:

Role of oxygen functional groups in reduced graphene oxide for lubrication

Bhavana Gupta[1,2], Niranjan Kumar[1], Kalpataru Panda[3], Vigneshwaran Kanan[1], Shailesh Joshi[4], Iris Visoly-Fisher[2]

[1]Materials Science Group, Indira Gandhi Centre for Atomic Research, Kalpakkam, India

[2]Department of Solar Energy and Environmental Physics, Swiss Institute for Dryland Environmental and Energy Research, Jacob Blaustein Institutes for Desert Research, Ben-Gurion University of the Negev, Midreshet Ben-Gurion 8499000, Israel

[3]Department of Advanced Materials Science, Graduate School of Frontier Sciences, The University of Tokyo, 5-1-5, Kashiwanoha, Kashiwa, Chiba 277-8561, Japan

[4]Radiological Safety Division, Indira Gandhi Centre for Atomic Research, Kalpakkam, India

Correspondence and requests for materials should be addressed to:
N.K. (niranjan@igcar.gov.in)

Content:

Figure S1. FTIR absorption spectroscopy and contact angles of PEG200 and PEG600 on steel surface.

Figure S2. (a) FTIR spectra of (a_i) rGO_1-PEG200, (b_i) rGO_1-PEG600, (c_i) rGO_2-PEG200, and (d_i) rGO_2-PEG600. Schematic description of the coupling of PEG with (b) epoxy-hydroxyl rGO_2 and (c) hydroxyl functionalized rGO_1 sample.

Figure S3. Raman spectra of (a_i) rGO_1-PEG200, (b_i) rGO_1-PEG600, (c_i) rGO_2-PEG200, and (d_i) rGO_2-PEG600 samples.

Figure S4. Load dependent friction coefficients of (a) neat PEG200 and (b) neat PEG 600; tribology test parameters: sliding speed 4 cm/s, ball: 100Cr6 steel.

Figure S5. Friction coefficients of (a) rGO_1 and (b) rGO_2 additives in PEG200 at various concentration; tribology test parameters: load: 1 N, sliding speed: 4 cm/s, ball: 100Cr6 steel.

Figure S6. Friction coefficients of (a) rGO_1 and (b) rGO_2 additives in PEG200 at various concentration; tribology test parameters: load: 5 N, sliding speed: 4 cm/s, ball: 100Cr6 steel.

Figure S7. Friction coefficients of (a) rGO_1 and (b) rGO_2 additives in PEG600 at various concentration; tribology test parameters: load: 1 N, sliding speed: 4 cm/s, ball: 100Cr6 steel.

Figure S8. Friction coefficients of (a) rGO_1 and (b) rGO_2 additives in PEG600 at various concentration; tribology test parameters: load: 5 N, sliding speed: 4 cm/s, ball: 100Cr6 steel

Figure S9. Wear width analysis of (a) pure PEG200 and PEG600, (b-c) rGO_1 and rGO_2 dispersed in PEG200 at 1 and 5 N, respectively, (d-e) rGO_1 and rGO_2 dispersed in PEG600 at 1 and 5 N, respectively.

Figure S10. Optical images of selected wear tracks and ball scars damages: (a-d) rGO_1 and rGO_2 additives blended PEG200 at 1 N and 5 N loads, (e-h) rGO_1 and rGO_2 additives blended PEG600 at 1 N and 5 N loads; tribology test parameters: rGO concentration: 0.1 mg. mL^{-1}, sliding speed: 4 cm/s, ball: 100Cr6 steel, sliding distance: 100 m.

Tribological properties of neat rGO.

Figure S11. Friction coefficients of steel-steel sliding interface, and of steel-steel interface with rGO_2 and rGO_1 deposited layer in the wear track; tribology test parameters: load: 5 N, sliding speed: 4 cm/s, ball: 100Cr6 steel.

Figure S12. Raman spectra of the steel surface (spot (a)) and on the wear track at various locations (spots (b-d)); tribology test parameters: rGO_2 concentration 0.1 mg/mL, load: 1 N, sliding speed: 4 cm/s, ball: 100Cr6 steel.

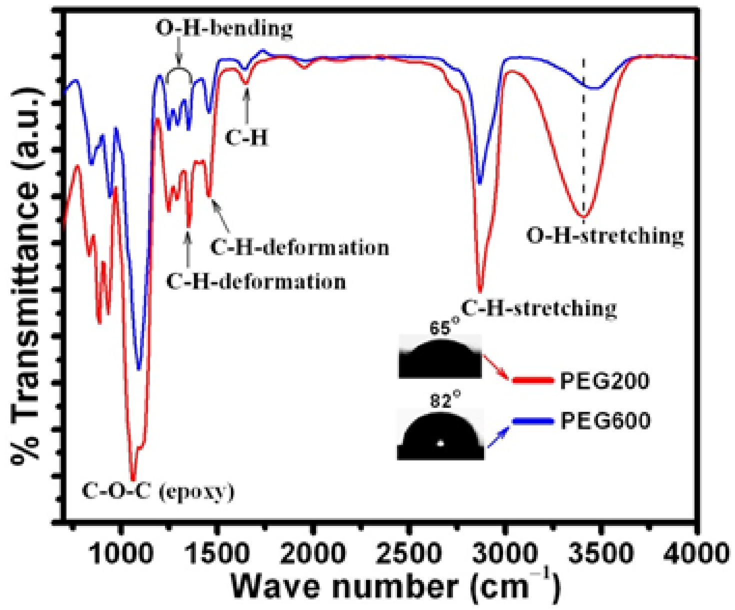

Figure S1. FTIR absorption spectroscopy and contact angles of PEG200 and PEG600 on steel surface.

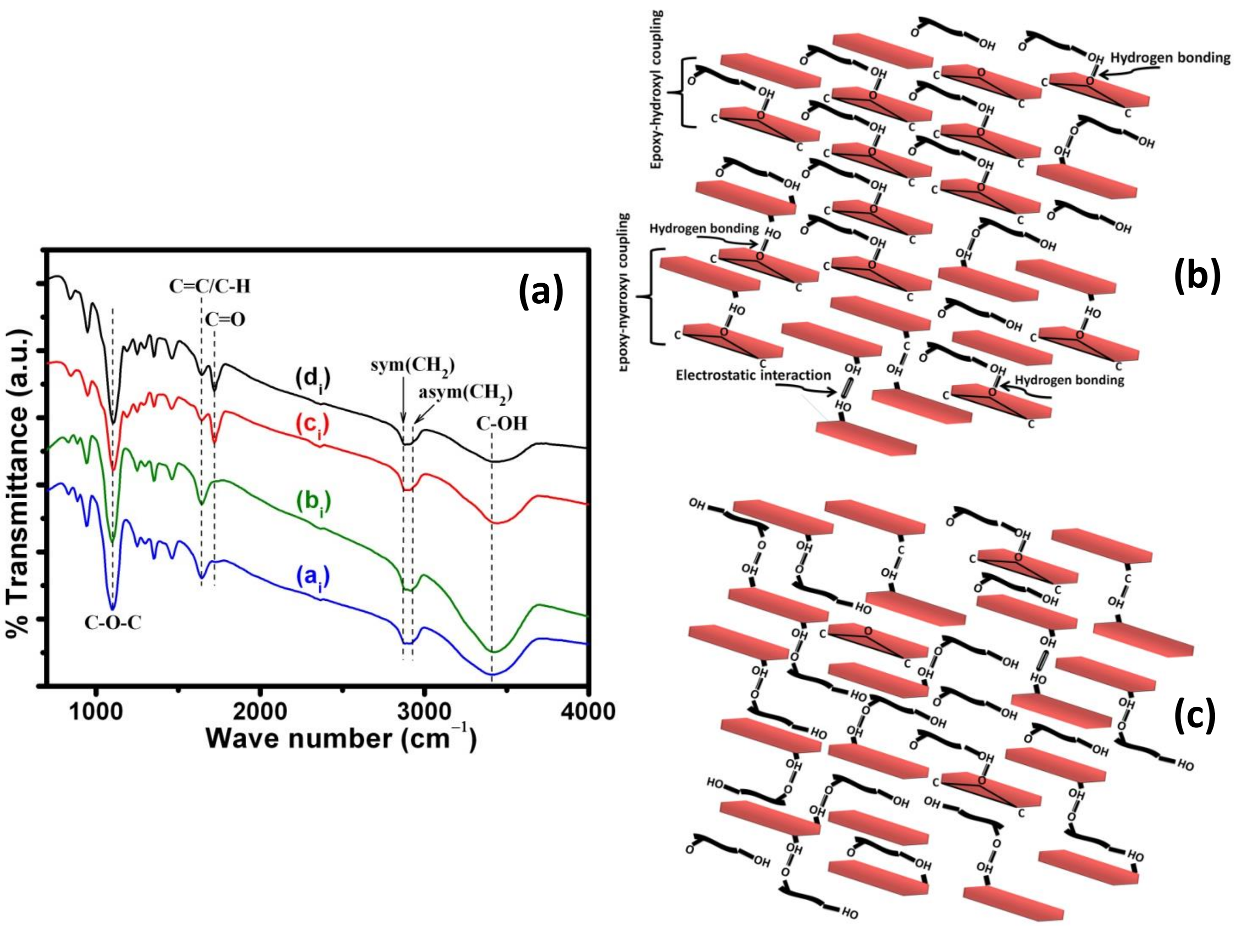

Figure S2. (a) FTIR spectra of (a_i) rGO_1-PEG200, (b_i) rGO_1-PEG600, (c_i) rGO_2-PEG200, and (d_i) rGO_2-PEG600. Schematic description of the coupling of PEG with (b) epoxy-hydroxyl rGO_2 and (c) hydroxyl functionalized rGO_1.

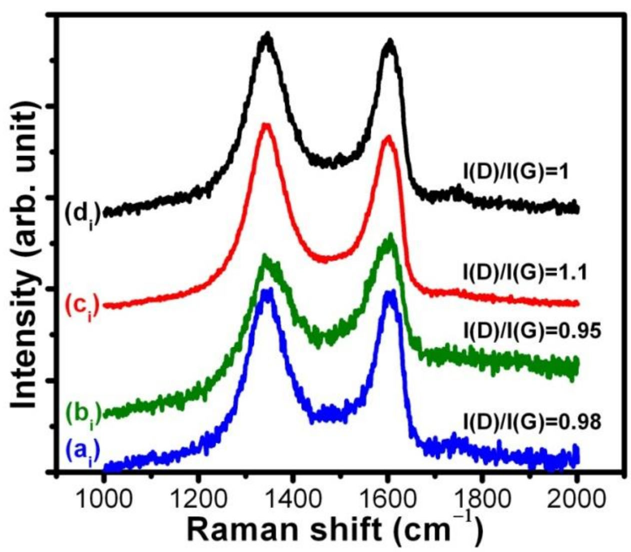

Figure S3. Raman spectra of (a_i) rGO_1-PEG200, (b_i) rGO_1-PEG600, (c_i) rGO_2-PEG200, and (d_i) rGO_2-PEG600 samples.

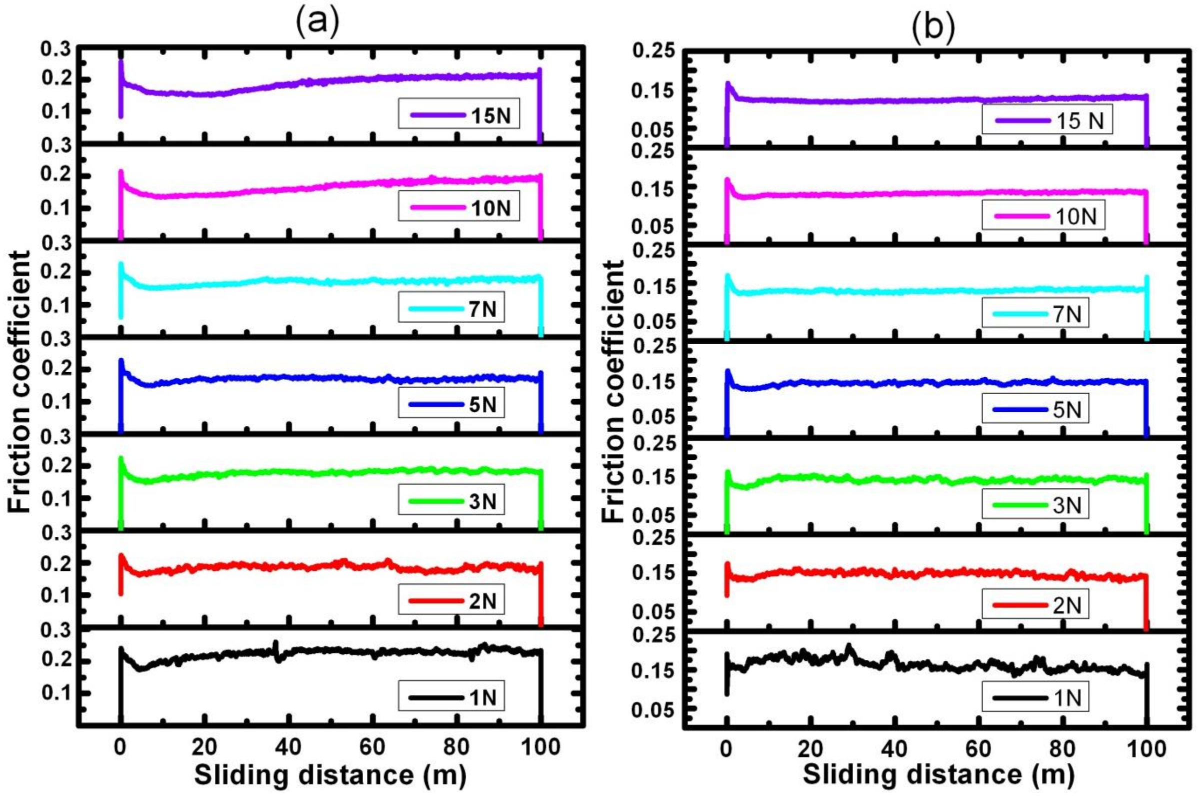

Figure S4. Load dependent friction coefficients of (a) neat PEG200 and (b) neat PEG 600; tribology test parameters: sliding speed 4 cm/s, ball: 100Cr6 steel.

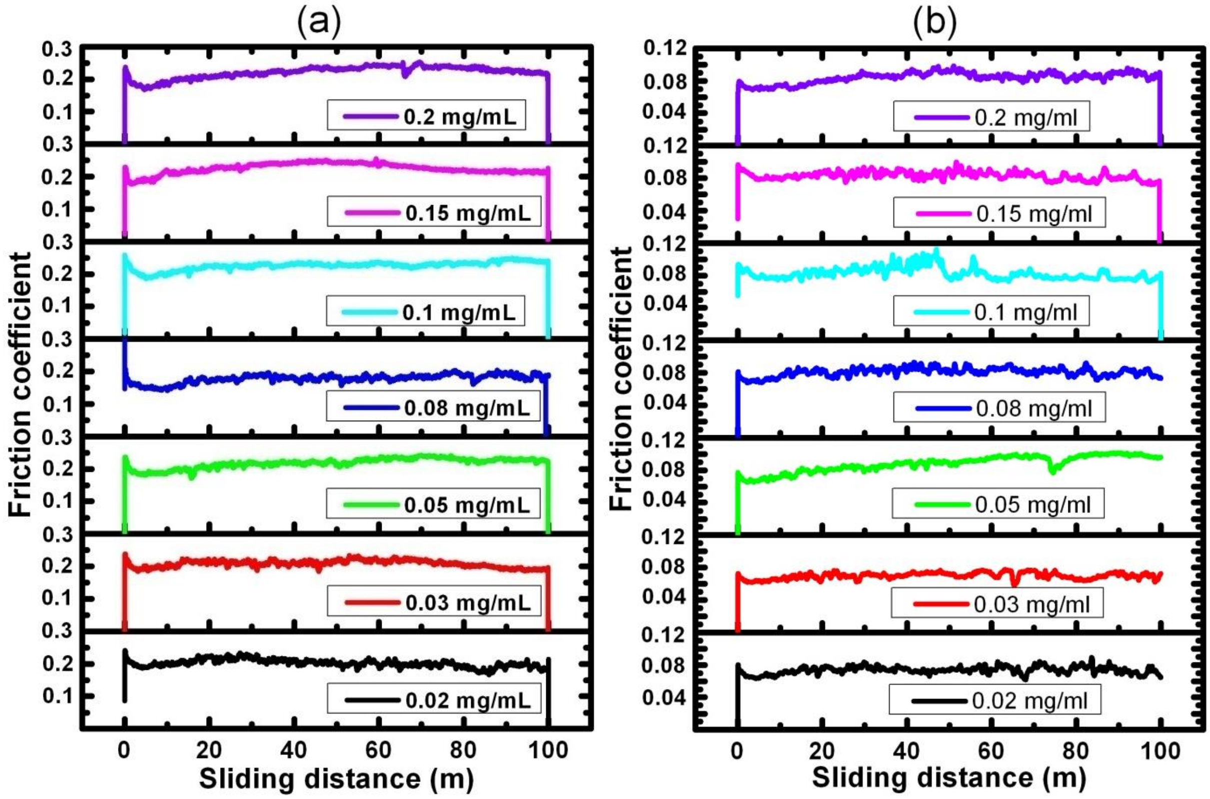

Figure S5. Friction coefficients of (a) rGO_1 and (b) rGO_2 additives in PEG200 at various concentration; tribology test parameters: load: 1 N, sliding speed: 4 cm/s, ball: 100Cr6 steel.

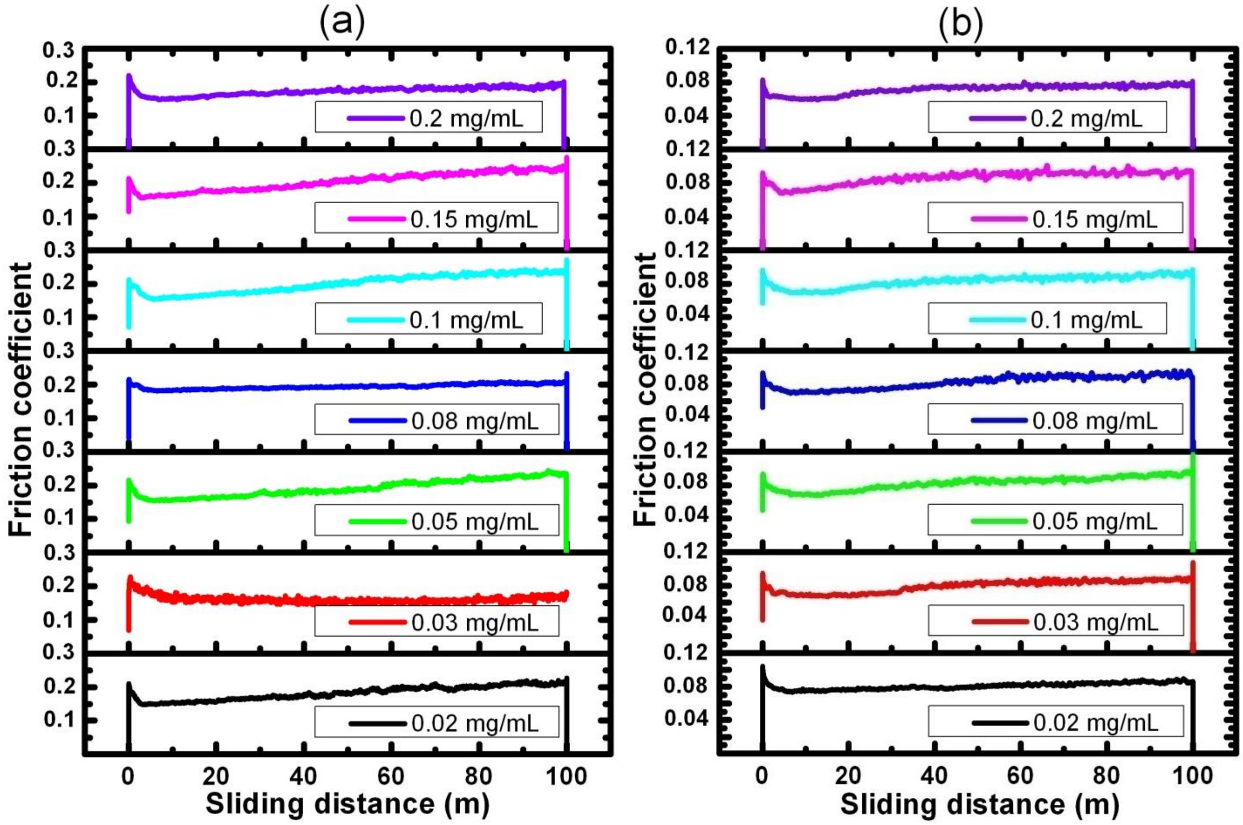

Figure S6. Friction coefficients of (a) rGO_1 and (b) rGO_2 additives in PEG200 at various concentration; tribology test parameters: load: 5 N, sliding speed: 4 cm/s, ball: 100Cr6 steel.

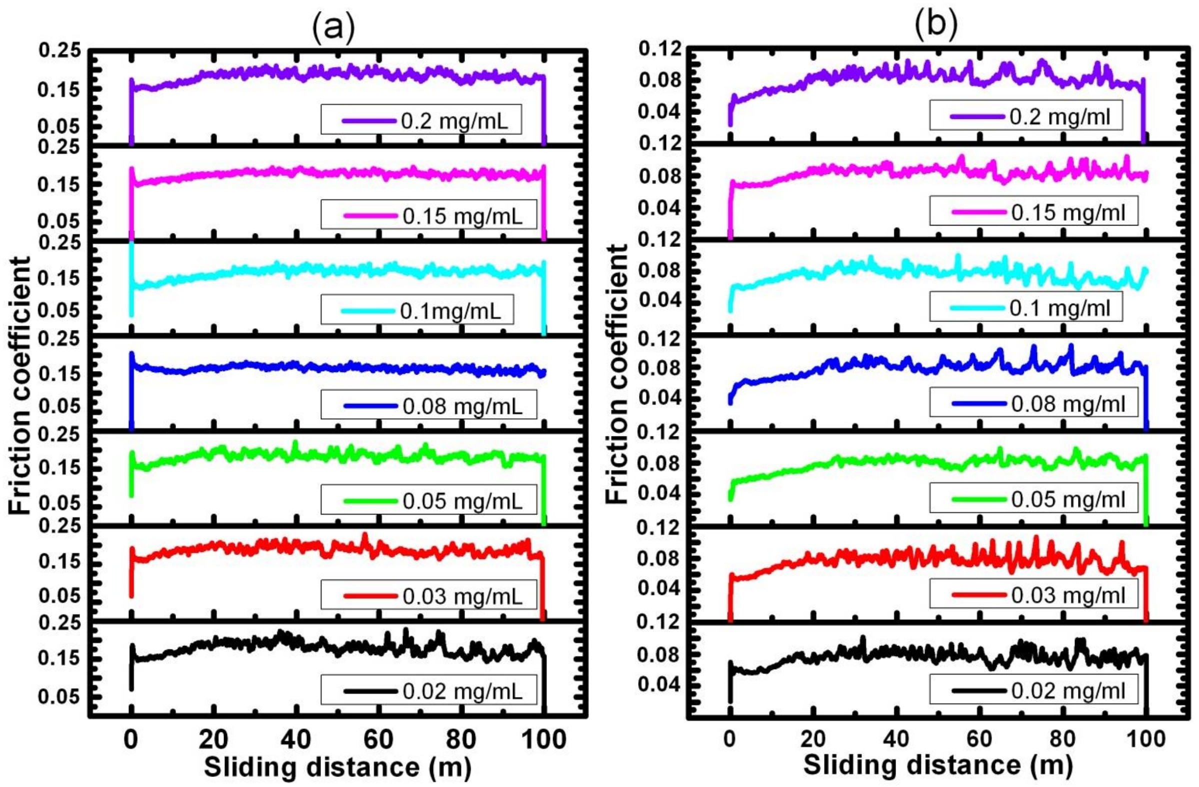

Figure S7. Friction coefficients of (a) rGO_1 and (b) rGO_2 additives in PEG600 at various concentration; tribology test parameters: load: 1 N, sliding speed: 4 cm/s, ball: 100Cr6 steel.

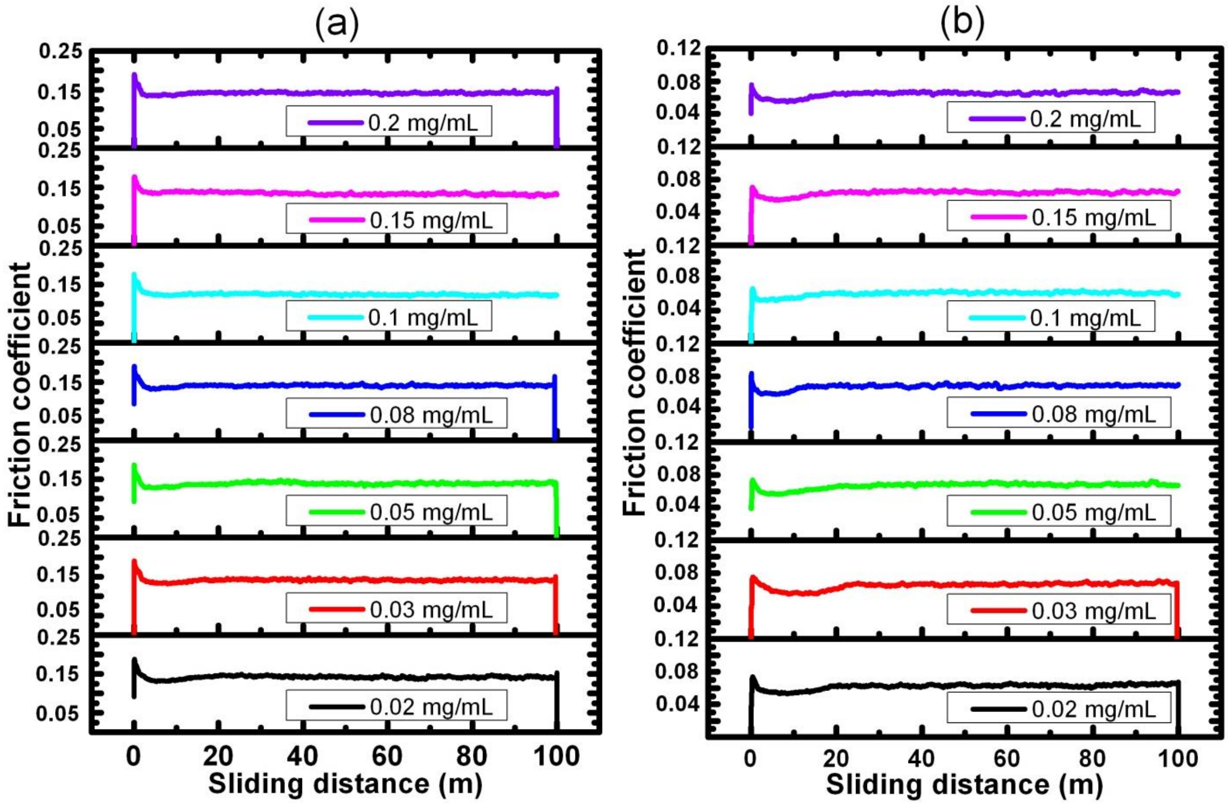

Figure S8. Friction coefficients of (a) rGO_1 and (b) rGO_2 additives in PEG600 at various concentration; tribology test parameters: load: 5 N, sliding speed: 4 cm/s, ball: 100Cr6 steel.

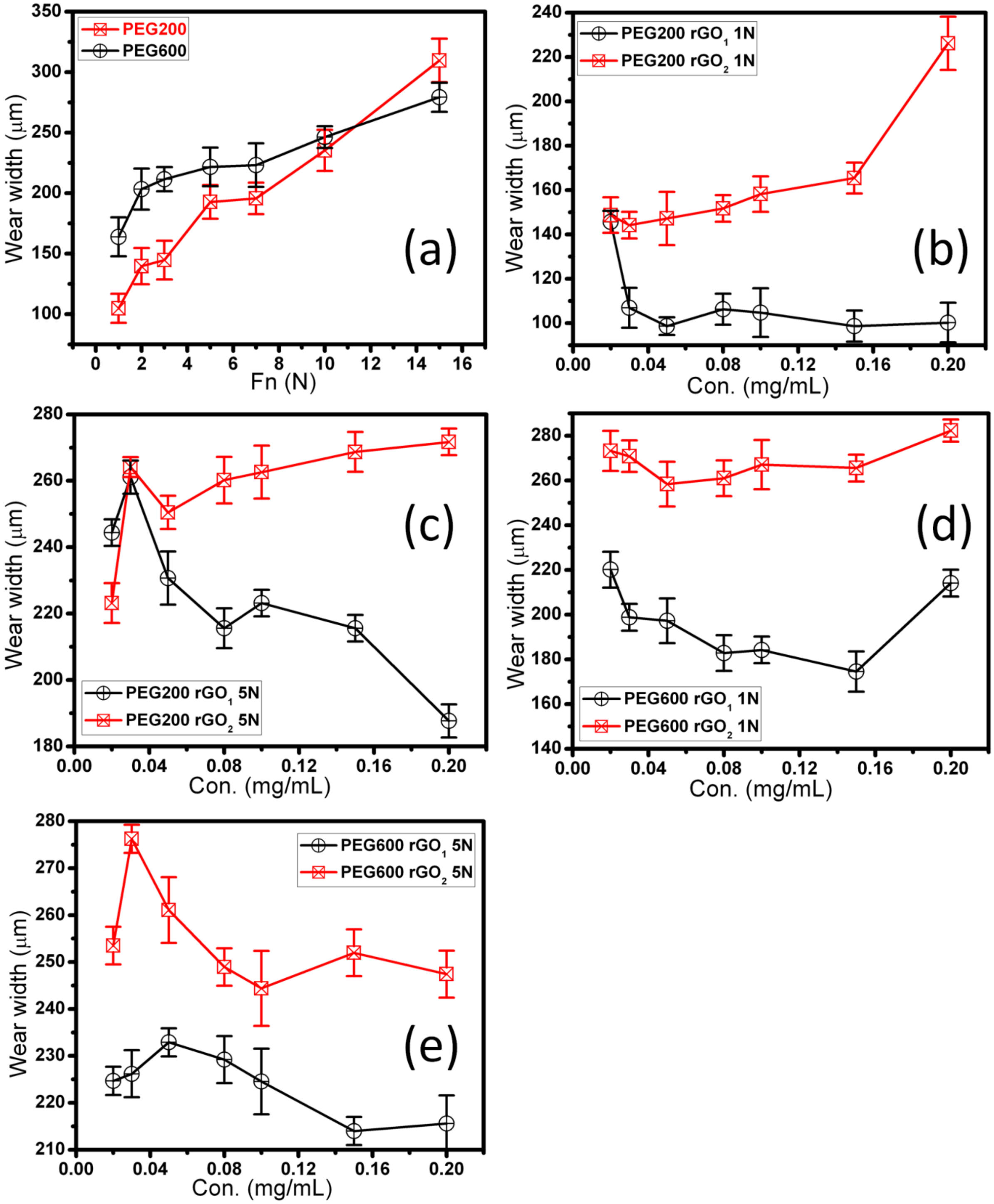

Figure S9. Wear width analysis of (a) pure PEG200 and PEG600, (b-c) rGO_1 and rGO_2 dispersed in PEG200 at 1 and 5 N, respectively, (d-e) rGO_1 and rGO_2 dispersed in PEG600 at 1 and 5 N, respectively.

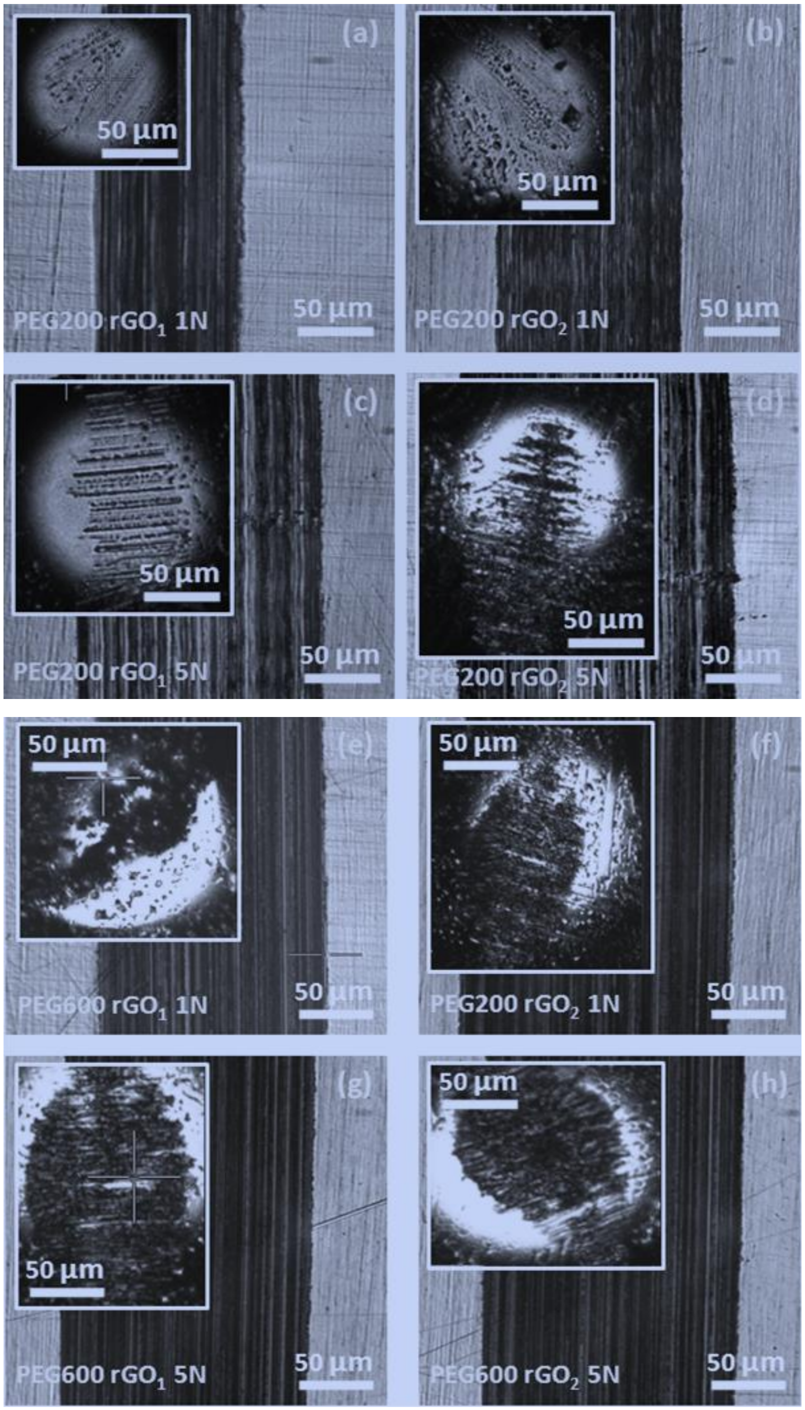

Figure S10. Optical images of selected wear tracks and ball scars damages: (a-d) rGO_1 and rGO_2 additives blended PEG200 at 1 N and 5 N loads, (e-h) rGO_1 and rGO_2 additives blended PEG600 at 1 N and 5 N loads; tribology test parameters: rGOconcentration: 0.1 mg. mL^{-1}, sliding speed: 4 cm/s, ball: 100Cr6 steel, sliding distance: 100 m.

Tribological properties of neat rGO.

The epoxy- and epoxy-hydroxyl-terminated neat rGOs were tested in macroscopic tribology test conditions to verify the results of the atomic scale model of friction mechanism proposed by Wang *et al.*[1]. For this experiment, the wear track was formed on a polished bare steel substrate sliding against a steel ball. The friction coefficient of the dry steel-steel, non-lubricated sliding contact is shown in the first part of the curve where the average friction coefficient is high (1.2) and it is unstable (Fig. S11). After this, the test was paused for a moment, and the wear track was cleaned; next a thick layer (approximately 2 μm) of the rGO_2 sample was deposited, and the test was run in the same track. Under these conditions, the friction coefficient dropped to the average value of 0.12. After this, the test was again paused, and the wear track was cleaned; next a 2μm layer of the rGO_1 sample was deposited on the wear track. Under this condition, the friction coefficient significantly decreased to an average value of 0.06. Here, the experimental conditions and contacting bodies were identical to those with the rGO/PEG blends. The friction coefficient of the epoxy-hydroxyl-terminated rGO_2 sample showed a higher value (0.12) due to the strong hydrogen bonding between the graphene sheets, in agreement with Wang *et al.*[1]. However, lower friction coefficient observed for epoxy-hydroxyl-terminated rGO_2 sample blended with PEG is attributed to the interaction of PEG with the graphene-oxide sheets.

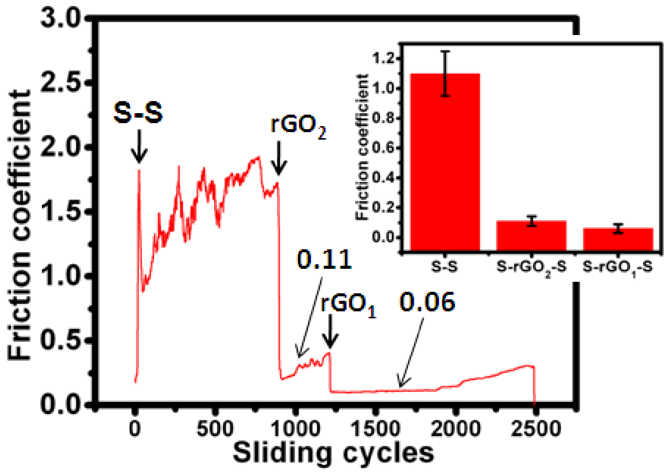

Figure S11. Friction coefficients of steel-steel sliding interface and steel-steel interface with rGO_2 and rGO_1 deposited layer in the wear track; tribology test parameters: load: 5 N, sliding speed: 4 cm/s, ball: 100Cr6 steel.

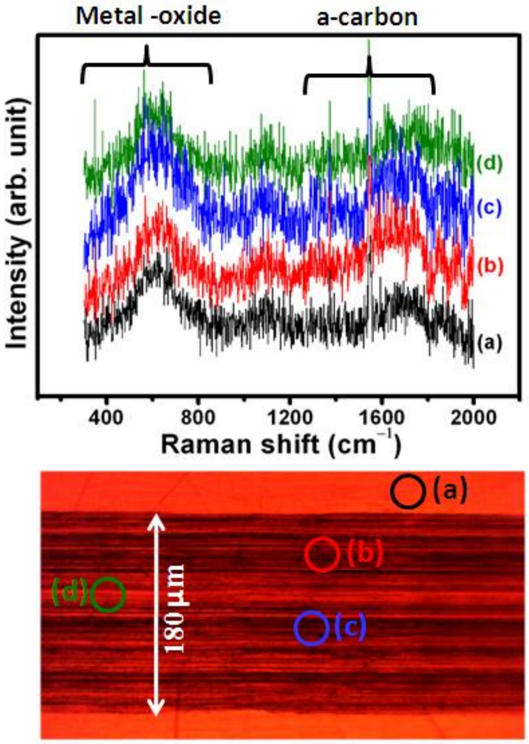

Figure S12. Raman spectra of the steel surface (spot (a)) and on the wear track at various locations (spots (b-d)); tribology test parameters: rGO_2 concentration 0.1 mg/mL, load: 1 N, sliding speed: 4 cm/s, ball: 100Cr6 steel.

Reference

1. Wang, L. F. *et al.* Atomic-scale friction in graphene oxide: an interfacial interaction perspective from first-principles calculations. *Phys. Rev. B.* **86**, 125436–125436 (2012).
